# Expert opinions on knowledge-translation interventions for occupational therapists working with neonates in South Africa: A Delphi study

**DOI:** 10.4102/hsag.v27i0.1724

**Published:** 2022-02-08

**Authors:** Samantha J. York, Gina Rencken, Michael O. Ogunlana, Ayesha Dawood, Pragashnie Govender

**Affiliations:** 1 Private, Samantha Campbell Occupational Therapy, Durban, South Africa; 2 Department of Occupational Therapy, Faculty of Health Sciences, University of KwaZulu-Natal, Durban, South Africa; 3 College of Health Sciences, University of KwaZulu-Natal, Durban, South Africa; 4 Department of Physiotherapy, Federal Medical Centre Abeokuta, Ogun State, Nigeria; 5 Department of Occupational Therapy, General Justice Gizenga Mpanza Regional Hospital, Durban, South Africa

**Keywords:** at-risk infant, childhood development, consensus methodology, Delphi study, early public health sector, knowledge translation, multidisciplinary team, neonatal care, occupational therapy

## Abstract

**Background:**

There is a paucity of literature on knowledge translation (KT) interventions for occupational therapists (OTs) in assessing and caring for the neonate and at-risk infant. Care at this stage of life is paramount, requiring a shift from the survival of the neonate, to the quality of survival. Consequently, clinicians working with neonates have a crucial role in ensuring optimal development and preventing long-term adverse developmental outcomes.

**Aim:**

This study aimed to explore experts’ opinions on KT interventions for OTs working with neonates and at-risk infants in South Africa.

**Setting:**

This study was located in South Africa. Due to the virtual nature of data collection, no geographical limitations within the country were imposed.

**Method:**

A two-round Delphi study with a multidisciplinary expert panel (*n* = 20; *n* = 18) was conducted. The round one survey was developed based on a literature review, findings from a preceding focus group, and a pilot study. The subsequent round was based on the data and comments generated from the first round. Results were pooled and presented to participants following both rounds.

**Results:**

Consensus on 127 items out of 130 was achieved. These included consensus on the definition of KT in neonatal care, the knowledge that OTs should possess, professional competencies, skills required, professional values, and characteristics. Further agreement was reached on the KT process, the usefulness of KT modalities, recommended courses in neonatal care, barriers to KT, best-practice and requirements for undergraduate training.

**Conclusion:**

Knowledge translation required for OTs working with neonates and at-risk infants were established in this study.

**Contribution:**

This study may be useful for consideration in contextually relevant KT interventions for clinicians working in neonatal care.

## Introduction

The first 28 days of an infant’s life, known as the neonatal period, are reported as the most vulnerable as they pose many health risks (World Health Organization 2018). Care at this stage of life is paramount and crucial, requiring a shift from only survival of the neonate to the quality of survival (World Health Organization 2018). Consequently, clinicians working with neonates play an important role in ensuring optimal development and preventing long-term developmental problems (Johnson 2017).

Evidence-based practice and specialised training are essential components that provide occupational therapists (OTs) the foundation to advocate and engage in developmental care in neonatal intensive care units (NICUs) (Legendre et al. 2011). There however, remains limited knowledge on whether occupational therapy (OT) undergraduate programmes adequately prepare OTs for practice in the NICU (Hardy, Govender & Naidoo 2021).

Johnson (2005) emphasised that being grounded both in knowledge transfer concepts and the knowledge translation (KT) process will lead to more effective exchanges and ultimately enhance therapists’ translation of evidence into practice. To ensure improved neonatal care, KT must be efficient and optimal. The OTs in South Africa (SA) have been utilising research while practising in the care of neonates and at-risk infants (Lecuona et al. 2016; Perks, Rencken & Govender 2020); however, there is limited evidence on the application of stakeholder-driven KT interventions for OTs in SA (Govender 2021).

When insufficient information on a topic exists, consensus methodology is often used (Hasson, Keeney & McKenna 2000). In this article, the authors report on a Delphi study that aimed to gather expert opinions on KT interventions specifically for OTs working with neonates and at-risk infants in the public health sector in SA.

## Materials and methods

### Design

**FIGURE 1:**
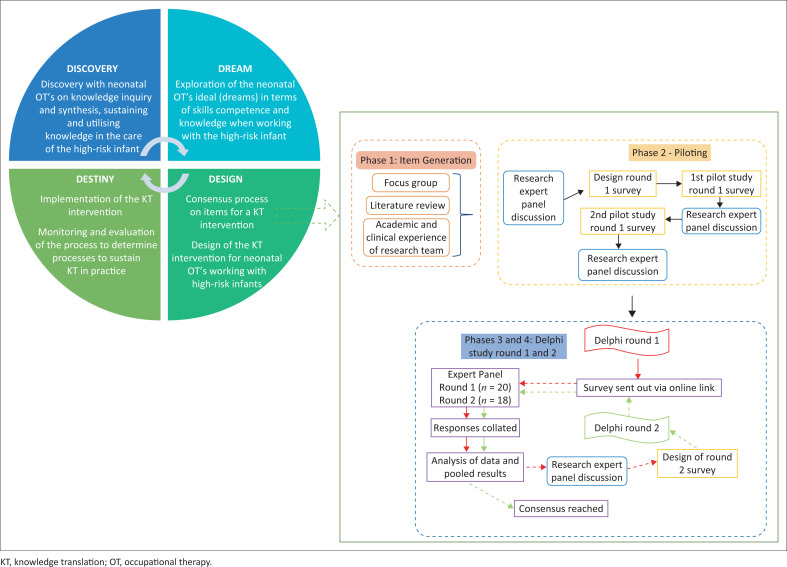
Study design (Delphi process) illustrated within the larger study design using appreciative inquiry.

This study forms part of a more extensive study on KT interventions for neonatal therapists using an appreciative inquiry process and is embedded within the ‘design’ phase of the process (Figure 1). A two-round Delphi technique, preceded by a rigorous appraisal of the literature, a focus group (Dawood et al. 2022) and a pilot survey, was used (Figure 1). The focus of this study was on the stability of the group consensus. A questionnaire was developed, uploaded onto Google forms, and participants were required to respond using a survey design, with ratings in the form of a Likert scale (Likert 1932; Salutini et al. 2020).

#### Phase one (item generation)

- **Focus group**: A preceding study on appreciating and envisioning knowledge needs in OT intervention for neonates in the public health sector of KwaZulu-Natal (KZN), contributed to the item generation of the survey (Dawood et al. 2022). Core themes that were derived and considered in the development of the Round 1 survey included: information on how knowledge was acquired and synthesised, how knowledge was utilised and translated, contextual barriers and adaptation, and what the ideal OT looked like in a neonatal setting.
- **Literature review**: A comprehensive literature review was also conducted. Google scholar was used to source evidence using key words such as, ‘neonatal care’, ‘NICU’ ‘occupational therapy’, ‘knowledge translation’. The search string included the following: (‘infant, newborn’ [MeSH Terms] OR [‘infant’ {All Fields} AND ‘newborn’ {All Fields}] OR ‘newborn infant’ [All Fields] OR ‘neonatal’ [All Fields] OR ‘neonate’ [All Fields] OR ‘neonates’ [All Fields] OR ‘neonatality’ [All Fields] OR ‘neonatals’ [All Fields] OR ‘neonates’ [All Fields] AND ‘care’ [All Fields] OR [‘intensive care units, neonatal’ {MeSH Terms}] OR ‘intensive’ [All Fields] AND ‘care’ [All Fields] AND ‘units’ [All Fields] AND ‘neonatal’ [All Fields] OR ‘neonatal intensive care NICU’ [All Fields] OR ‘nicu’ [All Fields] AND [‘occupational therapy’ {MeSH Terms} OR ‘occupational’] [All Fields] AND ‘therapy’ [All Fields] OR ‘occupational therapy’ [All Fields]) AND [‘translational medical research’ {MeSH Terms} OR {‘translational; (All Fields) AND; medical; {All Fields} AND ‘research’ {All Fields}] OR translational medical research’ [All Fields] OR [‘knowledge’ {All Fields} AND ‘translation’ {All Fields}] OR ‘knowledge translation’ [All Fields]).
- Studies undertaken in developed and developing countries were considered to understand best practices in high- and low-resourced areas. Neonatal care in SA was researched systematically, using keywords ‘occupational therapy’ and ‘knowledge translation’ to understand current practice and gaps in the literature.
- **Research team discussion**: Ensuing the focus group and literature review, items for the survey were roughly generated. This was presented to the research team (comprising of two paediatric academics/clinical educators/researchers [PG & GR], a post-doctoral research fellow/clinician based in sub-Saharan Africa [MOO], and a public sector clinician [AD] working in the field). The items were analysed, and the team contributed to the relevance, comprehensiveness, clarity, and completeness. The pilot study round one was subsequently formulated.

#### Phase two (piloting)

Pilot studies for Delphi surveys help identify ambiguities and improve the feasibility of administration (Powell 2003). For this study, experts were selected via purposive convenience sampling. All panellists had no less than three years’ experience in neonatal care and had attended training courses in neonatal care. A total of *n* = 10 pilot participants commented on the ease of use, the comprehensibility, and the time it took to complete the survey. Participants scored the relevance of each question (using *1 = not relevant, 2 = somewhat relevant, 3 = quite relevant, 4 = highly relevant)* as recommended by Polit and Beck (2006). The pilot study feedback was conveyed to the research team, interrogated, and relevant changes were effected. To ensure adequate psychometric properties, a second pilot study was undertaken. Five of the 10 pilot participants scored each item’s content validity index (Cabatan et al. 2020). This determined which items remained and which were said to be ‘irrelevant’ and were therefore removed from the questionnaire. This was discussed amongst the research team, and relevant changes were made.

#### Phase three (round one Delphi process)

*Selection and recruitment of participants:* Maximum variation purposive sampling and snowball sampling was used to recruit participants from clinical disciplines of paediatrics, nursing, OT, physical therapy (PT), speech and language therapy (SLT). Distinguishing experts is complex and ambiguous (Du Plessis & Human 2007; Hasson et al. 2000). Participants who had experience in the field of neonatal care for 2 years or more (Unsworth 2001), were registered with the relevant regulatory body (Health Professions Council of South Africa [HPCSA] or South African Nursing Council [SANC]), and/or had post-graduate training in neonatal care (Bruce, Langley & Tjale 2008), were recruited. As this study used a homogenous sample (all clinicians working in neonatal care), the sample size, although small, was considered and still yielded sufficient results (Skulmoski, Hartman & Krahn 2007). A list of 47 potential experts was formulated, and email invitations were sent out inviting participation. Each individual received a consent form, an information sheet and a link to the round one survey. Expertise was documented in a demographic section of the survey.

*Round one survey:* Each item in the questionnaire required panellists to rate their level of agreement between 1 (strongly disagree) and 10 (strongly agree) on items (Chyung et al. 2017). The items are demonstrated in each of the tables that appear in the results section of this article. Panellists were sent a reminder after 1 and 2 weeks. An *a priori* threshold of ≥70% for consensus was determined before data collection (Graham, Regehr & Wright 2003; Naidoo & Joubert 2013; Perks et al. 2020).

### Data analysis

#### Rankings

Descriptive statistics were used to analyse the data using Statistical Package for the Social Sciences (SPSS) version 21 (IBM SPSS Statistics for Windows 2020). The extent to which each participant agreed with the stated issue and the level of agreement between each other (descriptive statistics) (Naidoo & Joubert 2013) was ascertained. Cronbach’s alpha was also used to determine the internal consistency of the group response. Participants’ degree of agreement/disagreement was rated on a 10-point scale, where 1 = strongly disagree, and 10 = strongly agree. Data were summarised so that each response between 1 and 5 was given a value of 1 to indicate disagreement and each answer between 6 and 10, was given a value of 2 to indicate agreement. Percentages of agreement and disagreement were calculated.

#### Qualitative comments

The responses from Round one were analysed using content analysis and assisted in (1) additions and application to the Round two questionnaire and/or (2) comment for general consideration. Similar items were grouped and reported. The content analysis and the statistical summaries contributed to the development of the Round two survey.

#### Phase four (round two Delphi process)

Analysis and feedback from participants prior to Round two included a summary table with the percentages of agreement on each item. Items that had reached a consensus in Round one were excluded in Round two. The items that did not achieve consensus were included in Round two to allow participants to re-vote. Consensus was reached at this round.

### Ethical considerations

The UKZN Biomedical Research Ethics Committee (approval number: BREC/00001886/2020) approved the study. Ethical principles adhered to in the study included informed consent and autonomy, confidentiality (responses were collated anonymously using an identifying number known only to the authors) and voluntary participation (participants could withdraw from the study with no consequences).

## Results

### Demographic profile of panellists

The multidisciplinary sample comprised *n* = 20 participants in Round one and *n* = 18 in Round two. Panellists hailed from six out of nine provinces in SA. Experience ranged from 2 to 28 years. Of the 20 panellists, 17 had completed post-graduate courses in paediatric-related fields. Three participants who had not completed post-graduate courses were included in the study, based on having worked clinically for a minimum of 2–3 years in the field (Unsworth 2001). Two panellists functioned within an academic setting (Table 1).

**TABLE 1:** Demographic profile of panellists (*n* = 20).

Participant	Professional group	Age band	Gender	Neonatal care experience (in years)	Highest level of education	Geographic location (province in SA)	Post-graduate courses in neonatal care
P1	SLT	31–40	F	12	Masters	Gauteng	Certification in Neonatal Therapy, NBAS, Advanced NDT Baby), Little Steps, Training through NANT
P2	OT	31–40	F	10	Masters	Gauteng	NANT Ignite programme, NBAS, Prechtl’s GMA Advanced Practice for the complex neonate, Advanced NDT (baby), Certified Neonatal Therapist (NTNCB), Infant SI
P3	Midwife	41–50	F	28	Doctorate	Free State	Clinical Masters’ degree
P4	Professional nurse	41–50	F	26	Diploma	KZN	Certificate in NICU Care
P5	OT	31–40	F	7	Masters	KZN	NDSC, Infant SI, Infant massage, Reflex integration training
P6	SLT & Audiologist	31–40	F	5	Doctorate	Gauteng	NDSC
P7	Professional nurse	41–50	F	25	Doctorate	Gauteng	Advanced Midwifery and Neonatology
P8	PT	21–30	F	8	Masters	Gauteng	NDSC, Neonatal Gold Online course, Paediatric and Neonatal assessment and management (DOH), HINE, Basic Life Support/CPR for Neonates, SI for Allied Healthcare (Meg Faure)
P9	Midwife	51–60	F	21	Bachelor	Free State	Diploma in Neonatal Nursing Science and NDSC
P10	OT	41–50	F	2	Masters	WC	Perinatal neuroscience, NDT (paediatric), Kangaroo mother care
P11	SLT	21–30	F	3	Bachelor	WC	None
P12	PT	31–40	F	10	Masters	Gauteng	Advanced NDT (Baby), MSc Physio (Paediatric neonatal neurology), TIMP, Prechtl’s GMA, HINE, numerous lectures and short courses.
P13	Midwifery lecturer	41–50	F	20	Doctorate	North West	Advanced midwifery (include neonatal nursing), Ignite, NBAS, Baby Massage
P14	SLT	31–40	F	4	Bachelor	Gauteng	None
P15	OT	31–40	F	12–14	Bachelor	KZN	NDT (paediatric)
P16	Doctor	31–40	F	8	Masters	KZN	None
P17	SLT	21–30	F	7	Bachelor	Gauteng	Multiple skills building, online courses/webinars; Little steps neurodevelopmental supportive care of the preterm infant; Advanced NDT (baby)
P18	Professional nurse	51–60	F	25	Doctorate	Gauteng	Diploma in NICU nursing
P19	OT	41–50	F	17	Masters	KZN	Advanced NDT (Baby)
P20	OT	21–30	F	3	Bachelor	EC	None

GMA, General Movement Assessment; HINE, Hammersmith Infant Neurological Examination; NANT, National Association of Neonatal Therapists; NBAS, Neonatal Behavioural Scale; NDT, Neurodevelopmental Therapy; NDSC, Neurodevelopmental Supportive Care; SI, Sensory Integration; TIMP, Test of Infant Motor Performance; OT, occupational therapy; SLT, speech and language therapy ; PT, physical therapy; KZN, KwaZulu-Natal; WC, Western Cape; EC, Eastern Cape; NICU, neonatal intensive care units; CPR, cardiopulmonary resuscitation; SI, sensory integration; NTNCB, Neonatal Therapy National Certification Board; SA, South Africa.

Similar to other SA studies using the Delphi technique to achieve consensus on clinical practice issues, this study used a two-round Delphi (Naidoo & Joubert 2013; Perks et al. 2020). This was because of the consensus being reached after Round one on all but three sets of items.

### Delphi rounds and results

The Delphi Round one survey comprised 123 items. Seven items were added to the questionnaire for Round two, based on comments made by experts in Round one. Of a total of 130 final items in Round two, consensus was reached for 127 items. The three items not reaching consensus did not warrant an additional Delphi round. The findings are presented against each section with a percentage of agreement achieved in each round.

### Knowledge translation definition

The Round one survey included nine items related to the *definition of KT* specifically for OTs working in neonatal care. Consensus was achieved on all items. Experts recommended an additional two items (‘KT occurs within a system of interactions among the family’ and ‘KT aims to optimise enablers’). These items were included in Round two to which the experts agreed with the inclusion (Table 2).

**TABLE 2:** Definition of knowledge translation for occupational therapy working in neonatal care.

Round 1 *n* = 20 (%)		Round 2 *n* = 18 (%)		Statements for inclusion in the definition of knowledge translation for OTs working in neonatal care
Agree	Disagree	Agree	Disagree	
95	5	*	*	KT is a complex and dynamic process
85	15	*	*	KT involves attaining (verb that means reaching or achieving a goal) evidence
95	5	*	*	KT involves obtaining (to take ownership of something and is unrelated to any level of difficulty) evidence;
100	0	*	*	KT involves synthesising (identifying, selecting and combining results from multiple studies) evidence
100	0	*	*	KT involves exchanging (collaborative problem solving between researchers and decision-makers that happen through linkage – resulting in mutual learning) evidence
-	-	94	6	KT occurs within a system of interactions among the family
100	0	*	*	KT occurs within a system of interactions among a multidisciplinary team
-	-	94	6	KT aims to optimise enablers
100	0	*	*	KT aims to overcome various barriers to evidence utilisation
100	0	*	*	KT strategies should include adaptations to the local context
100	0	*	*	KT aims to apply the best possible care

KT, knowledge translation; OT, occupational therapy.

* Consensus reached in Round 1.

The final definition reads as, the:

[*C*]omplex and dynamic process which involves attaining, obtaining, synthesising and exchanging evidence, within a system of interactions among the family and multidisciplinary team to optimise enablers, overcome potential barriers to evidence utilisation and adapt to the local context to implement the best possible care for the at-risk infant and family.

### Professional knowledge of occupational therapy

Consensus was reached on all 14 items on the *knowledge that OTs should possess on specific diagnoses and interventions*. Further exploration of knowledge, for example, understanding ‘medical complications’, reached consensus on 11 out of 15 items. Four items (‘medical treatment protocols’, ‘effects of medication’, ‘respiratory support’ and ‘nutritional support’) had an agreement of ≤65% and were included in Round two. The question was also re-worded for Round two (from ‘has a knowledge on ()…’ to ‘should have knowledge on ()…’) to provide further clarity to experts and therefore all items were included again in round two. Consensus was reached on all 15 items in Round two.

### Knowledge translation process and knowledge brokerage

The necessity of a *knowledge broker (KB)* and the process of *knowledge brokerage* achieved 100% agreement following Round one. Consensus was also reached on all three items related to the *KT process* in Round one (Table 3).

**TABLE 3:** Knowledge translation process and knowledge brokerage (*n* = 20).

* Round 1 (%)		KT process and knowledge brokerage
Agree	Disagree	
100	0	**KT Process** Building partnerships between researchers, OTs, managers, and academics within the local context is important in the knowledge translation process for therapists working with at-risk infants
95	5	The organisation or managers within the organisation (within the public sector facility) play an important role in supporting the process of knowledge translation regarding the care of the at-risk infant
100	0	National therapy associations should contribute to knowledge translation through the support of peer-reviewed journals, position papers, guidelines, conferences and workshops and through resources and information on the website/page and in their newsletters
100	0	**Knowledge Brokerage** A knowledge broker (KB) is necessary in the KT process
100	0	A KB should include a ‘champion/broker’ in the facility that looks for KT opportunities
100	0	KB should include joint positions between universities and clinical settings to encourage exchange of information between clinicians and researchers for the development and translation of research
95	5	KB should include paediatric interest groups for OTs
95	5	KB should include paediatric interest groups for Ots

* Consensus reached in Round 1

KT, Knowledge translation; OT, occupational therapy; MDT, multidisciplinary team.

### Knowledge translation modalities

Concerning the effectiveness of KT modalities, an agreement of 100% was achieved on all 12 items in round one (Table 4).

**TABLE 4:** Knowledge translation modalities for the knowledge translation process (*n* = 20).

* Round 1 (%)		KT modalities for the KT process
Agree	Disagree	
100	0	Making use of multiple sources of evidence
100	0	Clinical experience
100	0	Internet (journal articles, websites)
100	0	Workshops (profession specific)
100	0	Workshops (multidisciplinary team)
100	0	Mentorship
100	0	Communities of practice (different interest groups, small-large associations)
100	0	In service training with other members of multidisciplinary team
100	0	In service training and journal reviews
100	0	Following knowledge acquisitions, consider context and create protocol
100	0	Following knowledge acquisition, consider context and update protocol (if there is already protocol in place)
100	0	Engage in a reflective process

KT, knowledge translation.

* Consensus reached in Round 1.

### Courses

In Round one, not all 20 participants rated every item, the responses varied from 16 to 18. Three of the listed courses, namely, ‘Little Steps Neurodevelopmental supportive care of the preterm infant 4-day course’ and ‘1-day course’ and ‘Movement Analysis Education Strategies (MAES) Therapy’ had an agreement of ≤67%. These were included in Round two and an additional five courses as recommended for inclusion by experts. These included ‘Special interest webinars or online courses’, ‘lactation support – Lactation consultant course SA’, ‘Neuroscience for Improved Neonatal Outcomes (NINO)’, Training in administration of ‘test of motor infant performance’ and ‘NDT/Bobath Advanced baby course (post foundation course)’. Of these eight items (three from round one and five additional items), consensus was reached on six items. After Round two, a consensus was not achieved for ‘MAES therapy’ and ‘lactation support consultant course SA’.

### Barriers to knowledge translation

Following Round two, only one item, ‘lack of financial incentives or promotion opportunities’ did not reach a consensus (Table 5).

**TABLE 5:** Barriers to knowledge translation.

Round 1 *n* = 20 (%)		Round 2 *n* = 18 (%)		Barriers to KT
Agree	Disagree	Agree	Disagree	
80	20	*	*	Lack of time to train
80	20	*	*	Lack of time to integrate knowledge into practice
60	40	72	28	Lack of available evidence
75	25	*	*	Lack of confidence in ability to integrate evidence into practice
75	25	*	*	Lack of clinical relevance in training
55	45	61	39	Lack of financial incentives or promotion opportunities
90	10	*	*	The organisation of the healthcare system (public sector health facilities)
75	25	*	*	Lack of existing recommended standards of practice
95	5	*	*	Individual healthcare professionals and their lack of knowledge
80	20	*	*	Attitudes in critically appraising and using evidence-based practice
85	15	*	*	Skills in critically appraising and using evidence-based practice

KT, knowledge translation.

* Consensus reached in Round 1.

### Best practice for neonatal care

All items on best practice reached an agreement of ≥ 95% in Round one and were hence precluded from Round two (Table 6).

**TABLE 6:** Best practice for neonatal care.

* Round 1 (%)		Best practice for neonatal care
Agree	Disagree	
100	0	Controlling environmental variables (noise, light etc.) to promote neurodevelopment
95	5	Daily multidisciplinary team interaction to discuss patient care
95	5	Flexible time to care for infants throughout the day
100	0	Time dedicated for family intervention
100	0	Integration of infant into the family unit
100	0	Ensuring a follow up multidisciplinary action plan
100	0	Monitoring early childhood development for first 3 years of life (high-risk baby clinic)
100	0	Ensuring infant safety, adaption and development
100	0	Making use of standardised assessments to monitor the progress of the infant (e.g. General movements assessments, Hammersmith Neonatal Neurological Examination [HNNE])

* Consensus reached in Round 1.

### Knowledge translation in undergraduate training

All items on KT for undergraduates reached a ≥ 95% agreement in Round one and were precluded from Round two.

### Reliability and validity

Conducting research in a team strengthens the research carried out within a Delphi study (Du Plessis & Human 2007). The additional input from the different researchers in the questionnaire development contributed to verifying the data analysis and promoted the validity of the items generated. The rigorous instrument development process using a literature review, focus groups and research team discussions allowed large amounts of data to be collected and ensured the data was rich and robust (Du Plessis & Human 2007). The pilot study and panel review also ensured that questions were well-phrased and easy to understand to improve reliability and validity (Hasson et al. 2001). The fact that this study used two successive rounds of the Delphi process also helps in increasing the concurrent validity (Hasson et al. 2000).

## Discussion

A two-round Delphi process, with a multidisciplinary panel of South African clinicians experienced in the field of neonatal care, were useful in establishing consensus for the definition of KT, professional competencies, the KT process, the effectiveness of KT modalities, barriers that may impede KT, best practice and KT for undergraduate training.

### Knowledge translation

Knowledge translation is recognised in OT as a driving force to improve healthcare (Metzler & Metz 2010). Although the Canadian Institutes of Health Research (2019) coined the term ‘KT’ in 2000 and provided a definition specific to their context, there has been no extension of this definition of KT to particular areas within the practice in OT, let alone neonatal and early intervention care, specifically within a low-resourced context. The definition that the panellists agreed to on KT for neonatal care is aligned to the available literature on KT and considerations in the care of neonates. It is well known that KT is ‘complex’ (Graham et al. 2006). Existing medical and nursing models do not clearly explain how to systematically integrate research findings within a client-centred practice context (Craik & Rappolt 2003). Therefore, the definition can assist practitioners in understanding KT within the context of neonatal care in a low-resourced context. This will enable objective and maximum benefit for policy, practice and patients (Bennett & Jessani 2011), and could aid in creating a KT model specific to neonatal care. Theories, process models, and frameworks should be considered when developing, implementing or evaluating KT interventions (Tricco et al. 2016). Therefore, having a relevant definition could be a starting point in improving the understanding of KT and how to implement and evaluate KT in this field.

### Professional competencies

One of the issues highlighted within KT is the growing accumulation of evidence and practitioners’ ability to keep up to date (Graham et al. 2006). To identify, review, and select knowledge as recommended by Graham et al. (2006), one needs to understand this knowledge. It is therefore essential to understand the professional competencies required to work in the neonatal field. Working in neonatal care is highly specialised and requires trained professionals to use specialised skills, sophisticated medical procedures and technology to treat fragile infants (Barbosa 2013; Dewire et al. 1996; Hardy et al. 2021; Vergara et al. 2006). This is also highlighted in the Neonatal Therapy Core Scope of Practice (National Association of Neonatal Therapists 2014). Although the operational therapist is not treating the specific diagnosis medically, they need to recognise acutely ill or premature infants’ complex medical needs and vulnerabilities. Panellists were required to rate common diagnoses in the NICUs in SA, the most common being, prematurity, birth asphyxia and infection (Limpopo Initiative for Newborn Care 2013). Experts unanimously agreed that OTs needed to have this knowledge.

Procedures, protocols, precautions, and support systems are crucial for the occupational therapist to understand when working in the NICU. Adapting or structuring the environment to enhance function is a well-accepted OT approach. The occupational therapist also needs to have a holistic understanding of the infant and the different interventions to collaborate and provide an environment of developmentally supportive care (Vergara et al. 2006).

### Knowledge translation process

The importance of KT has been receiving increased attention in the literature, especially in healthcare (Graham et al. 2006; Metzler & Metz 2010). Bennett et al. (2016) recognised the inclusion of a ‘knowledge champion’ or broker as an enabler of KT for OTs, as did experts in this study, despite limited literature on that role within a low-resourced context. Currently, there are no South African studies that use a KB in KT within occupational therapy. This may prove difficult as this may need to be a position on its own and may require funding (Bennett et al. 2016), limits of which already exist in public hospitals in SA (Buchanan 2011). It may also comprise a clinician who is already working in the setting. The practical implication of this may be problematic as clinicians are already dealing with high caseloads and inadequate time to fulfil all duties (Hardy et al. 2021). The feasibility of the inclusion of this role in the public sector will need careful thought. It may require organisational support as well as OTs to move slightly out of familiar contexts.

### Knowledge translation modalities

All items were agreed as applicable by experts. Both tacit knowledge and explicit knowledge were embedded in this section (David, Poissant & Rochette 2012). Tacit knowledge is knowledge accumulated through experience and is not explicitly expressed. Although tacit knowledge appears simple, a complex interplay of knowledge and skill is apparent (Metzler & Metz 2010). It is recommended that clinicians be given time/opportunities to share their knowledge with their team or other clinicians. Knowledge translation interventions at public hospitals should encourage communities of practice or organising forums where clinicians can share their experiences and learn from each other (Metzler & Metz 2010). Communities of practice (different group’s interest groups, small-large associations) could be challenging to implement practically because of time constraints and the ability to connect in KT activities with other clinicians (Bennett et al. 2016; Skulmoski et al. 2007).

### Courses

Not all clinicians are exposed to the same courses and are primarily based on their specific profession. For example, the MAES therapy course is open to various clinicians (doctors, speech and language therapists [SLTs], OTs, physical therapists [PTs]). However, most participants are either OTs or PTs (MAES Therapy 2021). This could be because of the focus of the course being on hands-on therapy rather than a medical approach which may most likely be more applicable to a medical doctor. The lactation consultant course also did not receive consensus. Although many OTs are practising in lactation, they have traditionally not considered breastfeeding within the scope of their profession in the public health sector (Visser et al. 2016). The highest consensus reached was on the course ‘IGNITE: Core Training and Mentoring Program for Neonatal Therapists’. This is an international course for OTs, PTs and SLTs that runs over 10 months. Although effective, this may be inaccessible to a large population of OTs because of financial reasons and time constraints. This is supported by recent studies (Dawood et al. 2022; Hardy et al. 2021) that found OTs had insufficient training in neonatal care because of post-graduate courses being unaffordable and not receiving funding from their places of work to improve knowledge and skills.

### Barriers to knowledge translation

Knowledge translation is intended to consider the range of influences affecting incorporating knowledge into practice (Metzler & Metz 2010). Experts agreed on the many barriers to KT (Johnson 2005). The highest agreement amongst experts was ‘individual healthcare professionals and their lack of knowledge’. This is concerning as throughout literature, the importance of advanced skills and knowledge for the occupational therapist working in the NICU is highlighted (Hunter, Mullen & Dallas 1994; Hunter, Lee & Altimer 2015; Vergara et al. 2006).

After round two, a *lack of available evidence* was also recognised as a barrier. Further studies should explore whether the availability of evidence is the barrier or how to access evidence; appraisal and understanding of evidence also remains the barrier (Grimshaw et al. 2012). Although consensus on lack of incentives or promotion opportunities were not achieved, this has been identified as a barrier in other studies (Metzler & Metz 2010). These authors discussed organisations playing a role in promoting KT through financial incentives or subsidised training as OTs reported that lack of finances hindered KT efforts (Metzler & Metz 2010). In 2007, the HPCSA implemented a continuing professional development programme to improve the quality of care provided to patients. This entails clinicians participating in post-graduate training, courses and workshops to accumulate continuing education units (CEUs) per 12-month period, including ethics, human rights and medical law (HPCSA 2021). However, South African OTs stated that although these are mandatory, they are not always affordable for the therapists (Dawood et al. 2022).

*Lack of time* is consistently reported by OTs as a barrier, as was evaluating and applying research knowledge (Metzler & Metz 2010). According to Metzler and Metz (2010), a therapist’s knowledge, expressed through clinical judgement and reflection, are essential tools in identifying barriers and supports. Clinicians need to judge how best to identify potential barriers given their understanding of the context and available resources. Clinicians need to consider these obstacles when planning KT interventions. Removing obstacles at the level of the practice environment is often beyond the control of most practitioners; however, practitioners can influence factors affecting KT at the level of the person (Metzler & Metz 2010). These could include a lack of confidence and knowledge, and attitudes in critically appraising and using evidence-based practice. An improved understanding of KT, awareness of personal circumstances, and recent research related to these influences are critical to creating more effective KT strategies. If clinicians have a better understanding of KT, they can design KT strategies that allow adaptation to the local context (Johnson 2005; Metzler & Metz 2010).

### Best practice

The at-risk neonate is not well-adapted to the stressful environment of the NICU (Vergara et al. 2006), therefore as experts agreed, controlling environmental variables is integral to best practice. Experts also agreed that daily interactions to discuss patient care forms part of best practice. This is the standard that is included in the Norms and Standards for Essential Care (Limpopo Initiative for Newborn Care 2013), and is in line with literature that highlights the fragility of the neonate and their ability to change presentation daily (Hunter 1996; Vergara et al. 2006). The other agreed best practices are helpful for clinicians in the field, and therefore should be considered for their training with at-risk infants. They can compare their current practice with what experts regard as ‘best practice’ and identify potential gaps. Efforts can then be focused on implementing ‘best practice’ and ultimately enhancing therapy in this area.

### Undergraduate training

In their study, Hardy et al. (2021) ascertained that novice OTs felt underprepared for work in the NICU because of limited or no knowledge or skills from an undergraduate training level. Although practical implementation is complex, academic clinicians need to consider these results when considering undergraduate training for OTs. Although the need for advanced knowledge and skills has been cited in the available literature in SA, novice OTs are expected to work in the NICUs post-undergraduate studies. There is, therefore the need for adequate exposure to the NICU and at-risk infants at an undergraduate level. Knowledge translation for OT student practitioners has also been cited as essential (Govender & Mostert 2019). Understanding KT aids in the uptake of evidence into practice (Johnson 2005). In line with this, experts agreed that undergraduate training includes increased academic knowledge on KT models, increased practical examples of adapting knowledge to suit the local context and increased collaboration of the MDT in the undergraduate period.

## Conclusion

The two-round Delphi process described in this article was useful in establishing consensus on a definition of KT for OTs within the area of neonates and at-risk infants in the public health sector, professional competencies required of OTs, KT process for OTs, the effectiveness of KT modalities, the barriers that may impede KT, best practice for OTs working with neonates and at-risk infants in public health hospitals and practices included in undergraduate training. Considering that consensus on these factors has been ascertained for OTs, future studies could develop interventions based on these principles and aid in the practical implementation of these KT strategies in the NICUs. Moreover, monitoring and evaluation of KT may be implemented over time. The gap between ‘best practice’ and current practice and how to improve undergraduate training practically, should also be explored.
